# Two Domains to Five: Advancing Veterinary Duty of Care to Fulfil Public Expectations of Animal Welfare Expertise

**DOI:** 10.3390/ani11123504

**Published:** 2021-12-08

**Authors:** Katherine E. Littlewood, Ngaio J. Beausoleil

**Affiliations:** Animal Welfare Science and Bioethics Centre, Tāwharau Ora—School of Veterinary Science, Massey University, Private Bag 11-222, Palmerston North 4442, New Zealand; N.J.Beausoleil@massey.ac.nz

**Keywords:** Five Domains Model, animal welfare science, welfare enhancement, veterinary education, continued professional development, veterinarian, animal welfare, quality of life

## Abstract

**Simple Summary:**

Veterinarians are animal health experts. More recently, explicit references to veterinarians as animal welfare experts have proliferated. Veterinarians are ideally situated to act as animal welfare experts by virtue of their core work with animals, influence over owners, their roles in policy development, compliance, and monitoring, and as educators of future veterinary professionals. However, the discipline of animal welfare science has moved beyond a focus on nutrition and health towards an acceptance that the mental experiences of animals are the focus of welfare consideration. The Five Domains Model is a framework for assessing animal welfare and focuses on mental experiences arising from a broad range of impacts or opportunities. The Model can be used as a framework to integrate contemporary understanding of animal welfare science in veterinary curricula and improve welfare literacy within the veterinary profession.

**Abstract:**

Veterinarians are animal health experts. More recently, they have been conferred a leading role as experts in animal welfare. This expectation of veterinarians as welfare experts appears to stem from their training in veterinary medicine as well as professional contributions to welfare-relevant policy and law. Veterinarians are ideally situated to act as animal welfare experts by virtue of their core work with animals and potential influence over owners, their roles in policy development, compliance, and monitoring, and as educators of future veterinarians. However, since its inception as a discipline over 70 years ago, animal welfare science has moved beyond a two-dimensional focus on nutrition and health (biological functioning) towards an understanding that the mental experiences of animals are the focus of welfare consideration. The Five Domains Model is a structured and systematic framework for more holistically considering conditions that contribute to the animal’s internal state and its perception of its external situation, and the resultant mental experiences. The Model can be used to better align veterinary animal welfare expertise with contemporary understanding of animal welfare science and improve welfare literacy within the veterinary profession. Improved understanding of animal welfare science is likely to lead to increased confidence, competence, and empowerment to act as experts in their daily lives.

## 1. Introduction

As a result of their training, veterinarians hold primary authority and responsibility for animal health [1]. As an example, the World Organization for Animal Health (OIE) delegates the responsibility of implementing animal health and welfare measures to veterinarians in each member country; veterinarians are the only professionals designated ‘Competent Authority’ in the OIE’s Terrestrial and Aquatic Animal Health Codes [2,3]. Veterinarians are also called upon for expert commentary and knowledge of animal health during disease outbreaks e.g., [4,5] and are the first port of call for the treatment of sick or injured animals [6,7].

By virtue of their role as animal health experts, veterinarians have also implicitly been conferred primary expertise in safeguarding animal welfare more generally [2,3,6,7]. This view of veterinarians as the predominant animal welfare experts appears to stem from their training in veterinary medicine, as well as professional expectations outlined in national and international strategies and laws (e.g., [1,8]), and by veterinary regulatory bodies e.g., [9,10]. For example, part of the OIE’s approach to improving animal welfare globally is to provide guidance “*…to Member Countries in order to strengthen their Veterinary Services to enhance their capacity to implement animal welfare standards*” [8]. Added to this, the New Zealand Code of Professional Conduct for Veterinarians expressly mentions that “*Veterinarians have a special duty to protect animal welfare and alleviate animal suffering*” [9]. The result of these increasingly common explicit references is that veterinarians are now often regarded as experts in animal welfare in many contexts and have special legal and professional obligations [9,11,12,13]. In addition to these obligations, veterinarians have a ‘duty of care’ to the animals under their care [14,15,16]. This duty extends to their clients, as the owners of the animals they care for. In a broader sense, veterinarians, as professionals are held to account by the wider public. There is an expectation that veterinarians will act professionally and use their skills for the benefit of both animals and people [9,10,14].

While veterinarians are ideally placed to safeguard animal welfare, understanding of animal welfare, the ways of scientifically assessing welfare states, and expectations for animals’ welfare have changed significantly over the last five or six decades [17,18,19,20,21,22]. This evolution of animal welfare as a scientific discipline in its own right has resulted from advances in veterinary, medical, behavioural, psychological, neurological, cognitive, and animal sciences [20,23]. The result is that animal welfare is now characterised more broadly and includes consideration of the mental experiences of animals, that is, how they experience their situation and life [17,18,19,20,21,22]. Health and nutrition, the historical focuses of veterinary training, represent only two of the domains that are considered in a holistic appreciation of an animal’s welfare state [17,18,19,20,21,22]. In addition, there is a growing expectation that, to provide animals with good welfare, we must ensure that they have a wide variety of positive experiences, rather than simply eliminating negative experiences [17,18,19,20,21,22,24]. These changes in knowledge and expectations have led to greater expectations of veterinarians to safeguard and enhance animal welfare in broader ways.

This review explores the expanding role of veterinarians in protection and promotion of animal welfare. We begin with a brief history of the role and the ways in which veterinary science has contributed to advances in animal welfare science thinking. We then discuss how the traditional focuses of veterinary medicine, that is, health and nutrition, are now considered to be components of more holistic science-based understanding and assessment of animals’ welfare states. Finally, we make recommendations for how the veterinary profession can advance their implicit and explicit duty of care and take steps towards fulfilling their role as experts in animal welfare by embracing these animal welfare advancements to a greater extent in both training and professional practice. The scope of this review is limited to consideration of how contemporary animal welfare science (i.e., use of scientific methods to understand and assess the capacity of animals for, and elicitation of, mental experiences relevant to welfare) can be taught to, and used by, veterinarians. This is not to diminish the importance of training in related topics such as animal ethics, policy, and law for veterinarians, but instead aims to focus the reader’s attention on the potential for veterinarians to apply their scientific knowledge more broadly. Throughout, examples will be presented from a New Zealand context.

## 2. Advances in Scientific Understanding of Animal Welfare Have Changed the Way Welfare Is Characterised and Assessed Leading to Changes in Expectations for the Welfare of Animals under Human Care

The term ‘animal welfare’ is used to describe both an academic discipline and a feature of sentient animals. Animal welfare is a complex and multi-faceted subject that has scientific, ethical, economic, cultural, religious, political, and legal dimensions [8,17,18,19,20,21,22,25]. In addition, animal welfare is now generally considered to refer to the subjective state of an animal, as perceived by the animal itself, and is a representation of its overall mental experiences [24,25,26,27]. Quality of life is conceptualised as the animal’s welfare state (i.e., overall mental experiences) over a longer timeframe [27]. As we now consider the welfare of animals over time, quality of life and animal welfare have become synonymous terms [20,27]. Sentience is the capacity to have negative and positive mental experiences and is a feature of those animals about whose welfare veterinarians and welfare scientists are generally concerned [25,28]. In most jurisdictions, animals assumed to be sentient include all vertebrates and a few neurally and behaviourally complex invertebrate taxa e.g., [1,29] (Box 1).

Box 1 **Sentience in legislation.**

In 2015, The New Zealand Government amended the Animal Welfare Act 1999 to include explicit recognition of animal sentience [1]. This inclusion achieved two things for animal welfare in New Zealand: (1) it acknowledged that animals (as defined in the Act) have the capacity to experience positive and negative mental states; and (2) this acknowledgement resulted in the affective state orientation to understanding animal welfare being explicitly recognised by New Zealand law.

As a discipline, animal welfare science is inextricably linked to animal ethics and legislation by virtue of its foundation. In the 1950s and 1960s, ethical questions were raised about how animals were being treated in factory farming situations because of growing public awareness of such treatment [30,31,32]. As a result of a 1965 United Kingdom government report, recommendations were made for increased research in various scientific disciplines to improve our understanding of farmed animal’s needs [18,19,25]. From this beginning, interest in the welfare of animals has broadened to include animals in a range of contexts (e.g., wild animals in captivity, animals in research, companion animals), resulting in the evolution of a separate scientific discipline known as animal welfare science [18,19,25].

Veterinary and various animal sciences are amongst the many disciplines that have contributed to improving our understanding of animals’ needs [18]. The work done in the mid- to late- 1900s improved our understanding of, and ability to identify, prevent, treat, optimise, or otherwise manage aspects of animal nutrition, health, disease, and dysfunction, alongside increasing productivity in farmed animals. Therefore, one of the first roles of veterinarians in relation to animal welfare was as researchers providing the knowledge that would later be used to improve the lives of animals [18,19,25].

Veterinarians were positioned as animal welfare experts by virtue of their clinical role as animal health experts [33,34,35,36]. There was support for this view from the predominant orientation to understanding and assessing animal welfare at that time, that is, ‘biological functioning’ [18,19,37]. Proponents of this orientation put emphasis on the biological function or physical health of the animal when assessing welfare states. According to this orientation, ‘good’ animal welfare is considered to occur when the animal has good health, productivity, reproduction, and other such metrics of physical function [18,19].

During the 1970s and 1980s, this orientation to animal welfare was generally considered sufficient to assess the welfare status of animals. For animals in many situations, meeting their basic (‘survival-critical’) needs was not as easy as it is with today’s knowledge and technology [18,19]. Therefore, health and productivity were appropriate metrics for welfare when the overall welfare status of animals was poorer. However, with advances in scientific knowledge about animals’ biology and behavioural needs and their neurological and cognitive capabilities, came advances in our understanding of animal welfare. The now-dominant, ‘affective state orientation’ facilitates an understanding of animal welfare beyond biological functioning [17,18,19,25].

### 2.1. Extending the Focus of Animal Welfare to Include Consideration of Affective State

According to the affective state orientation, ‘good’ welfare exists when an animal experiences an overall positive mental (affective) state [18,19]. Affective states are mental experiences that have valence (i.e., are experienced as positive/pleasant or negative/unpleasant) and thus have significance to the animal [28,38]. That significance is proposed to confer a fitness advantage for sentient animals by motivating responses, both immediately and in the future, that protect the animal from harm (for negative experiences) or encourage it to engage with beneficial circumstances (for positives) [28,38]. Affective experiences arise due to neural processing of sensory information about the internal state of the animal (i.e., features of its health and physical function) but also reflecting its perception of its external environment [17]. In this regard, the affective state orientation integrates the biological functioning orientation in that physical state and mental state are dynamically inter-related.

With this new understanding it has become clear that, in some cases, animals can be healthy and productive but have poor welfare nonetheless and that evaluating physical state/biological function alone does not provide a holistic understanding of animal welfare. Nor can that approach alone facilitate what is now generally considered to be ‘good’ welfare. For example, in some cases, selective breeding of farm animals for productivity can result in a decline in an animal’s overall welfare status despite its physical state being apparently good.

### 2.2. The Five Domains Model for Assessing Animal Welfare Facilitates More Holistic Evaluation of Welfare States Including Positive Experiences

**Figure 1:**
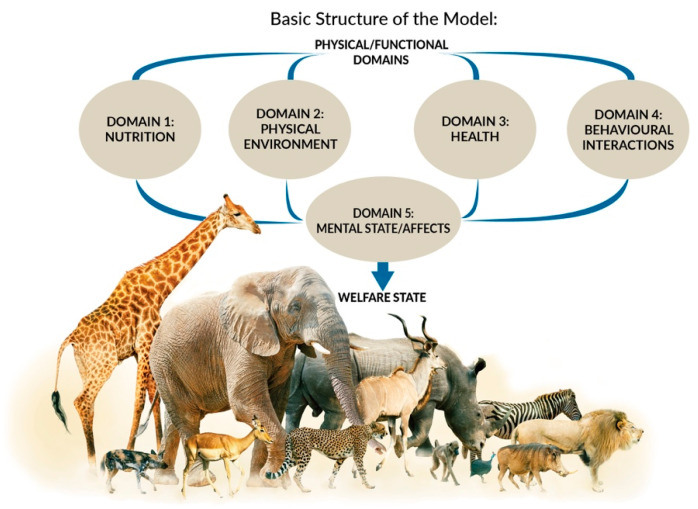
The 2020 Five Domains Model adapted by Cristina Wilkins from (Mellor et al. [17]).

As noted, animal welfare is now understood as the integrated mental experiences animals have as the result of their perception of their internal state and external situation [24,25,26,27]. The Five Domains Model (Figure 1) reflects this understanding and facilitates more holistic assessment of animal welfare states [17,37]. The Model is only one example of a framework that uses this approach to animal welfare assessment, but it has numerous advantages for the purposes of teaching and clinical practice. The Model encourages consideration of conditions that contribute to both the animal’s internal state and external situation, and the resultant mental experiences. The structure is based on evidence from neurophysiology, animal behaviour, veterinary and animal sciences, and other allied disciplines [17,21]. The Model comprises four physical/functional domains and a fifth, mental domain. Within the physical/functional domains, there are three internally driven ‘survival-related’ domains: Nutrition (food and hydration status), Physical Environment (e.g., thermal, noise, and space), and Health (disease, injury, and functional status). The fourth domain, Behavioural Interactions, represents the animal’s perceptions of aspects of its external situation, particularly its social environment [17].

Survival-related conditions are mainly aligned to our understanding of an animal’s basic biological functioning (e.g., food, shelter, and health). In contrast, behaviours resulting from the animal’s perception of its external situation can be interpreted with reference to behaviours expressed in natural environments or in situations where agency is promoted [17,18,19]. Agency refers to the animal’s engagement in voluntary, goal-directed behaviour; the ability to exercise agency results in ‘positive affective engagement’ when animals are given ‘freedom of choice’ [21,39]. Finally, by including the fifth Mental State domain to evaluate the experiences an animal may have because of the conditions in the four physical/functional domains, the Model explicitly focuses the user’s attention on the affective state orientation to animal welfare [17,18,19].

**Figure 2:**
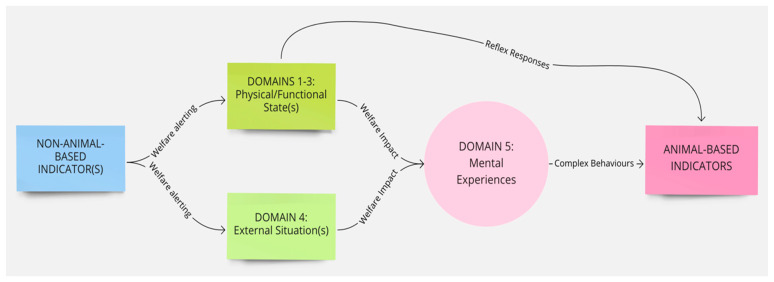
The logical link between physical/functional states can be used to validate animal welfare assessments using observable or measurable objective indicators applied to each of the first three physical/functional domains and their relationship to proposed mental experiences in Domain 5. Observable and measurable evidence of impacts and benefits is collated in Domains 1 to 4 and used to infer mental experiences in Domain 5. Validation of the link between situation-related experiences arising due to the animal’s perception of its external situation (Domain 4) is more challenging than for the link between physical/functional states (Domains 1 to 3) and proposed mental experiences.

Importantly, we are unable to directly measure an animal’s mental state, therefore, we rely on indirect assessments of mental experiences in Domain 5, i.e., inference based on observable/measurable indicators. Such inferences of mental experiences in non-human animals must be made with care [21,40,41]. Observable or measurable indicators of physical states and their relationships to the animal’s conditions or management and their proposed mental states need to be scientifically validated to avoid claims of anthropomorphism [41]. Briefly, animal-based (AB) indicators are preferred because, as output measures, they more directly reflect an animal’s likely mental experience [41] (Figure 2). However, in many cases non-animal-based indicators (NAB i.e., resource-based, and management-based) are acceptable because the link between these conditions and the animal’s physical state has unequivocally been demonstrated [41]. For example, prolonged absence of drinking water can be used to infer dehydration and thirst in species that need to drink regularly. Likewise, for some species and situations, there is detailed understanding of the neurological mechanism for generating a specific mental experience (e.g., thirst, pain, hunger, breathlessness) which is known to motivate or accompany an appropriate observable behavioural and/or physiological response [41]. For example, lack of water (NAB indicator) and the mental experience of thirst might motivate water-seeking behaviour (AB indicator). This link is more difficult to establish for situation-related mental experiences arising due to the animal’s perception of its environment because less of the underlying research for validating indicators and these linkages in Domain 4 has been done. Most of the AB indicators used in this domain are behaviours (Figure 2).

Advancements in our knowledge of animal welfare have allowed for such nuanced understanding about how physical/functional states (e.g., provision of adequate nutrition) can impact upon the mental experiences (e.g., satiety or hunger) of animals and therefore their overall welfare status. These advances have also allowed for an understanding of how situation-related conditions may impede animals’ ability to express strongly motivated behaviours or achieve important goals, i.e., their agency (e.g., barren environments) and result in negative welfare states (e.g., frustration or boredom) despite being in satisfactory physical condition.

## 3. Veterinarians Are Ideally Placed to Advance Animal Welfare in a Range of Contexts

Veterinarians are ideally placed to advance animal welfare in a range of contexts (Table 1) [12,13,37,42]. This potential results from their knowledge and training in veterinary science, their access to animals and their carers in a range of different contexts, their expert contributions to policy and law relating to animals and the public’s trust in them because of their status as medical professionals [9,10,14,43,44,45,46].

The combination of veterinarians being considered trusted professionals and their roles in society results in them being in situations of influence over animal welfare. Clinical veterinarians are responsible for the health and wellbeing of the animals under their care. This role is dictated as much by their training as by statements to this effect in Codes of Professional Conduct (e.g., [9,10]). Clinicians spend much of their day navigating interactions with their clients [14]. These interactions include recommendations about how animals are managed in a way that has the potential to improve their welfare. In this respect, veterinarians are welfare educators and information providers. Clinical veterinarians contribute towards protecting animal welfare (preventing or alleviating negative experiences) and have the potential to enhance the welfare of the animals under their care (promoting positive experiences).

As well as their primary role as clinicians, veterinarians work in a wide range of other fields and roles and thus have significant influence on animal welfare. For example, in many jurisdictions, veterinarians are employed as monitoring and compliance officers in slaughter premises where their role is to ensure animal products meet standards for the domestic market and those of export countries [42,47]. This involves verification of animal welfare and food safety requirements and certifying products for export [42]. They are animal welfare officers at research institutes and ensure research, teaching, and testing using animals is carried out in an ethical way [1]. They work for governmental bodies and are given special powers of authority in this role. As animal welfare inspectors, they may obtain and execute search warrants [1,48].

Importantly, veterinarians contribute, through their roles in government departments, to crafting or revising the very laws and standards that regulate our interactions with animals. Others are called upon to provide expert input to these processes, both nationally and internationally [2,3,49]. In addition, veterinarians are often called upon to act as expert witnesses in legal cases involving animal cruelty or neglect [50].

These specialized roles illustrate just some of the ways in which veterinarians are fulfilling their role as animal welfare experts beyond the scope of clinical expertise. Veterinarians are also employed as educators to train the next generation of veterinarians, veterinary paraprofessionals (e.g., veterinary nurses and animal scientists), and animal scientists. In this role, they are uniquely positioned to advance the expertise of future veterinarians in animal welfare.

Overall, veterinarians have roles in protecting animal welfare dictated by legal and professional obligations, by a duty of care for animals and their clients, and by society in general (Table 1). These roles place veterinarians in positions of responsibility and authority when it comes to animal welfare. Therefore, there are expectations that veterinarians are competent and confident to act as animal welfare experts.

## 4. Training to Improve Animal Welfare Literacy within the Veterinary Profession

The OIE Global Animal Welfare Strategy “*…supports the inclusion of animal welfare in curricula for veterinarians, veterinary paraprofessionals and students of animal agriculture and schools when relevant*” [8] To fulfil expectations of animal welfare expertise, veterinarians need to be knowledgeable, competent, and confident in their own skills and abilities [51,52].

As a result of the advancements in our understanding of animal welfare, expectations have changed for veterinarians. Animal welfare is no longer limited to considerations of biological functioning and animal health [18,19,25,33,34,35,36,37]. There is now an opportunity for veterinarians to engage more widely with animal welfare science and expand their focus to consider the mental experiences of animals arising from conditions in multiple domains [17,33,34,35].

In this next section, we discuss how the necessary skills and knowledge, that is competency, to assess animal welfare can be achieved through veterinary training initiatives. We first describe the current situation regarding veterinary training in animal welfare science, using the New Zealand veterinary science curriculum to illustrate, before moving on to discuss how veterinary curricula could be flipped to prioritise animal welfare science training and improve animal welfare literacy in Section 5.

### 4.1. Current Approach to Animal Welfare Science Training in Veterinary Curricula and Possible Consequences

In its current form, veterinary training is chiefly aimed at developing professionals with competencies in clinical sciences including pathology, pharmacology, diagnostics, and therapeutics. Because of this clinical focus, animal welfare science has thus far been presented as an isolated subject in most veterinary curricula [37,53]. In many programmes, welfare tends to be presented as a stand-alone subject in the earlier or pre-clinical years, either within or separate to other pre-clinical courses such as anatomy and physiology. As this is often the first exposure students have to the science of animal welfare, the teaching content is necessarily broad and theoretical and often taught as part of broader presentation of welfare-related topics like animal ethics, law, and behaviour [53,54]. In part, this may be because it is difficult to apply animal welfare science concepts to clinical examples when students do not have the necessary grounding in other related scientific disciplines. For example, they may not be able to extrapolate the welfare implications of delaying euthanasia of a terminally ill patient without understanding the pathophysiology of the disease, which is taught in later years [55,56].

By focusing animal welfare science teaching at only one (pre-clinical) location in veterinary training, and without any clinical context, students do not benefit from an integrated understanding of how they can apply animal welfare in practice. In our own experience, students taught welfare-related topics in first year fail to retain that understanding to the end of the degree and enter clinical practice with less welfare-literacy than would be desired. The failure to integrate and reinforce welfare as a key aspect of veterinary medical practice throughout the degree may implicitly communicate to students that it is of lesser importance than other, more clinical subjects, a perception that may be carried into professional practice. Thus, there is a need to integrate welfare science into every aspect of veterinary training or, in fact, to situate veterinary science within a broader fundamental understanding of welfare science.

#### 4.1.1. Pre-Clinical Teaching

The pre-clinical years of a veterinary degree aim to provide students with basic knowledge and skills in a range of core disciplines [37,53]. Students learn the normal structure and function of a range of animal species and are introduced to animal handling and animal needs in early animal welfare and behaviour subjects. There may also be disciplines such as biochemistry and farm systems taught in these early years.

At this stage, animal welfare is taught as a basic science and mostly with a theoretical focus. This is appropriate for the level, often low, of prior knowledge students have in the discipline. Students first need to know what animal welfare is, how it is understood and what it means for them, how it can be assessed, and basic features of animal use and husbandry procedures, for example, humane slaughter and pain management [37].

In our New Zealand context, the disciplines relating to animal welfare science align with a basic understanding of Domains 1 (Nutrition) and 2 (Physical Environment) with some understanding of normal animal behaviour aligning with Domain 4. Students are also introduced to some of the affective experiences (Domain 5) animals may have arising from these domains for example, pain, discomfort, hunger, and thirst.

#### 4.1.2. Para-Clinical Teaching

After learning the basics of veterinary science, students move on to consider pathology and pharmacology. In pathology, students are taught the causes and effects of disease processes. They build on their prior knowledge of normal anatomy and physiology to understand animal diseases [57]. Pharmacology is also introduced at this point, and students learn how drugs work on body systems and their impact on disease processes.

There is some overlap between clinical and paraclinical teaching. In the paraclinical, and sometimes preclinical, years students are taught about the treatment of a limited number of animal species and how to handle clinical cases from presentation through to diagnosis and treatment. They may have some exposure to cases in clinics, but much of the teaching at this stage is via lectures or case-studies.

This would be an excellent location for animal welfare science education to continue. It could be used to help students understand the impacts of disease processes and therapeutics on the overall welfare of the animals under their care. The established links between physical/functional states and likely mental experiences of the animal can be aligned to welfare indicators and then serve as useful revision of physiological control systems. Animal welfare science could be used to cement student knowledge and apply what they are learning to the whole animal. For example, students could explore the impact of anti-emetics used to treat the unpleasant experience of nausea associated with renal disease. This therapy could have positive impacts in both the nutritional (e.g., improving animal feed intake), health (reduced vomiting), and mental state (reduced nausea and malaise) domains. However, it may also have negative impacts in the behavioural interactions domain if the anti-emetic induces fatigue, thereby reducing the animal’s ability to interact within its environment, with other animals, and with humans. A nuanced, whole animal, understanding of veterinary medicine could result from inclusion of more animal welfare training at this stage in the degree.

#### 4.1.3. Clinical Teaching

Clinical teaching is defined here as ‘on the job’ or ‘in-hospital’ training [58,59]. This can range from one to three years, depending on the veterinary school [55]. At this stage in the degree, the focus tends to be on medical and surgical cases, that is, how to diagnose and treat diseases or pathologies. Preventative care training is often focused on vaccination, parasite control, body condition management (e.g., body weight and body condition scores), and dietary recommendations. In the case of production animals, this focus also includes herd health concepts.

Overall, current veterinary curricula provide students with vast knowledge of indicators aligning to Domain 3 (Health) and some skills in Domains 1 (Nutrition), but limited knowledge of indicators contributing to welfare assessments aligning to Domains 2 (Physical Environment) and 4 (Behavioural Interactions) [33,34,35]. As all four of these domains should be considered when assessing the overall welfare status of an animal in Domain 5 (Mental state), it follows that the current training paradigm does not provide veterinarians with sufficient knowledge and skills to be animal welfare experts. Instead, the current training paradigm produces two dimensional (Nutrition and Health) experts. We propose that to be experts in animal welfare, veterinarians must consider all five domains in the animals under their care. The next section illustrates how this could be achieved.

### 4.2. Aligning Para-Welfare Knowledge to Improve Veterinary Animal Welfare Assessments and Create Animal Welfare Experts

The current case-based focus of clinical training in veterinary medicine tends to limit animal welfare considerations to alleviation of negative experiences relating to just two domains (Nutrition and Health). This approach may also result in moral distress for veterinarians if a case is unsolvable and/or results in euthanasia of an animal under their care. If veterinarians are unable to evaluate multidimensional welfare impacts they may find it difficult to reconcile a euthanasia decision with the animal’s two-dimensional clinical picture [60]. Veterinarians do naturally assess the welfare of the animals under their care using indicators aligned to other domains, however, this process and the knowledge it leverages is not explicitly recognised. If animal welfare was embedded throughout the veterinary degree, students could more explicitly understand and articulate how they are evaluating welfare holistically and where the gaps in their assessments may be [37]. By way of example, veterinary students in their final year of study are often required to present a clinical scenario to their peers and teachers in a formal case rounds format (Box 2). Students use this opportunity to demonstrate their knowledge and understanding of a particular clinical syndrome or disease by explaining the aetiology, epidemiology, pathophysiology, and treatment outcomes for their chosen case. By taking this approach, the focus of these case presentations has been on the physical/functional state of the animal and particularly impacts in Domains 1 and 3. However, there is potential for a holistic animal welfare evaluation to be undertaken, with a focus on the mental experiences of the animal, by incorporating a comprehensive Five Domains assessment into these student presentations. By putting greater emphasis on what the animal itself experiences during clinical assessment and treatment, student clinicians might reach different conclusions about a treatment or euthanasia decision for an animal. This kind of integrated approach to animal health and welfare has the added benefit of cementing student understanding of animal welfare, improving animal welfare literacy, and allows them to act as experts in their future clinical work [37].

Box 2 **Veterinary student rounds case study.**

**Scenario:** A final year veterinary student is presenting a case of an 11-month-old cross breed dog that presented to the clinic for vomiting, lethargy, and inappetence. This dog was unvaccinated and had mild abdominal pain, hyperthermia, and grade 1 yellow faeces. A problem list and differential diagnoses are discussed and Parvovirus suspected. An ELISA antigen snap test was positive for Parvovirus. The student goes on to describe the pathophysiology of parvoviral infection in dogs, the treatment protocol (including intravenous fluid therapy, antibiotics, anti-emetics, and naso-oesophageal feeding tube placement), and concludes with the financial costs of treatment.

In this case study, details from the original presentation illustrate its alignment with the Five Domains Model. From this we can see that the original presentation focuses on Domains 1 (Nutrition) and 3 (Health), with only nominal mention of an affective experience (pain) aligned with Domain 5. Domains 2 (Physical Environment) and 4 (Behavioural Interactions) have not been discussed and the physical/functional state of the animal is also inadequately presented using a traditional case rounds format.

**DOMAIN 1: Nutrition**

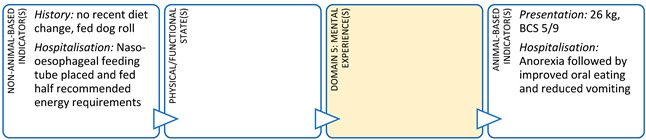

**DOMAIN 3: Health**

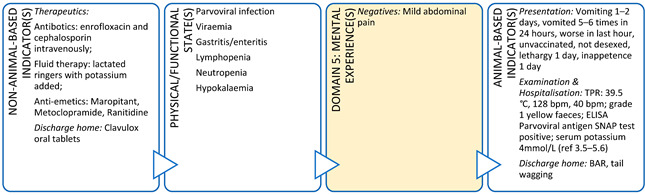

### 4.3. Continued Professional Development

In common with the clinical training of veterinary students, and for reasons already discussed, there is a need for practicing veterinarians to improve their animal welfare science literacy, with a particular focus on animal welfare assessments [37]. Some options already exist for New Zealand-based veterinarians to undertake continued professional development in animal welfare. The Australian and New Zealand College of Veterinary Scientists (ANZCVS) has an Animal Welfare Chapter with membership examinations offered every two years [61]. The University of Edinburgh offers a post-graduate certificate, diploma, or Master’s degree in International Animal Welfare, Ethics, and Law [62,63]. These two options have the advantage of being offered at a distance, and veterinarians can study towards the related qualifications in their own time. However, the learning outcomes for both options are more theoretical than practically focused, and those for the Edinburgh post-graduate qualifications are not specifically focused on veterinarians. There is a need for veterinary-specific animal welfare training for practicing veterinarians that is both practical and comprehensive. To achieve this, training should be co-designed alongside veterinary end-users (e.g., clinical veterinarians from a range of backgrounds and practice types, industry veterinarians, and those working as animal welfare officers) and with practical applications in mind.

A training initiative that focuses on practical use of animal welfare science for veterinarians is likely to improve animal welfare literacy, as well as empower veterinarians to act as experts in their daily lives. This training could also offer a means of operationalising the Five Domains Model for use by practicing veterinarians in a range of contexts. By way of example, Box 3 illustrates the potential role a clinical veterinarian can take to advance the welfare of dairy cattle during a routine herd health consultation and support their client at the same time. Many dairy cattle veterinarians evaluate grass cover as they drive through the farm gate to visit a cow or cows. Grass cover is a non-animal-based indicator of feed availability (quantity and quality) and can help alert a veterinarian to a problem if the cover is insufficient for the feed requirements of a dairy herd. In other words, low grass cover may alert a veterinarian to a potential nutritional inadequacy which may result in animals experiencing hunger in the future if the situation is not rectified. These implicit welfare assessments by veterinarians are performed during routine on-farm animal interactions but without locating them within the broader context of animal welfare. A more explicit approach would allow veterinarians to explain and justify their choice and interpretation of indicators of welfare and have more confidence in their welfare assessments and client recommendations.

In addition, a veterinarian in this context could use all five domains to make recommendations that could enhance the welfare of the animals under their care (Box 3). In discussion with the farmer (and illustrated using the Five Domains Model), a veterinarian can describe the importance of various welfare indicators (e.g., shelter/shade and resting areas – usually Domain 2, enrichment opportunities such as cow brushes, appropriate grazing management, positive human-animal interactions and good stockmanship) aligned to Domains 2 (Physical Environment) and 4 (Behavioural Interactions) that have the potential to provide for positive mental experiences and enhanced welfare for these animals. There is also scope for veterinarians to understand more about these welfare indicators, what they represent about the animal’s potential welfare status, and how directly they reflect this i.e., input (Box 3, left side: non-animal-based) versus output indicators or welfare (Box 3, right side: animal-based).

Box 3 **Production animal case study.**

**Scenario:** You are a large animal veterinarian who has a routine on-farm dairy cow herd health evaluation scheduled on an extensive (pasture-based) farm. This farm supplies milk to a company that pays a premium for milk from farms with an animal welfare plan. To obtain this premium, a veterinarian must help the farmer develop and implement this animal welfare plan. In preparation for your visit, your practice has developed a discussion framework based on the Five Domains Model.

This case study uses the Model to illustrate the welfare indicators that could be discussed with the farmer for physical/functional states (Domains 1 to 3) or external situations (Domain 4) and the potential welfare implications (mental experiences) these might indicate in Domain 5. Indicators more appropriate for veterinary-based welfare assessments have been identified with an asterix.

**DOMAIN 1: Nutrition**

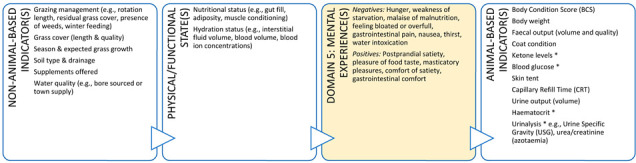

**DOMAIN 2: Physical Environment**

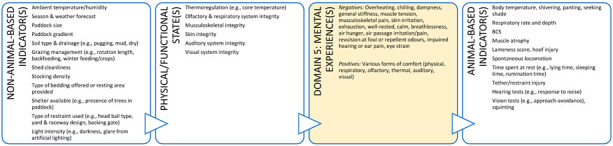

**DOMAIN 3: Health**

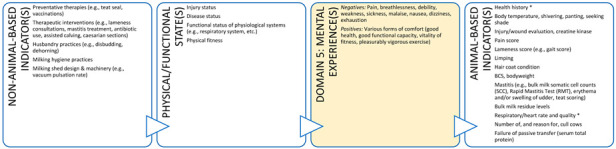

**DOMAIN 4: Behavioural Interactions (with the Environment)**

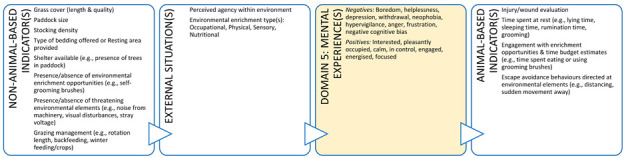

**DOMAIN 4: Behavioural Interactions (with Other Animals)**

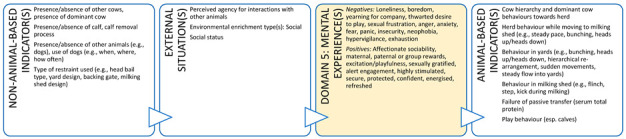

**DOMAIN 4: Behavioural Interactions (with Humans)**

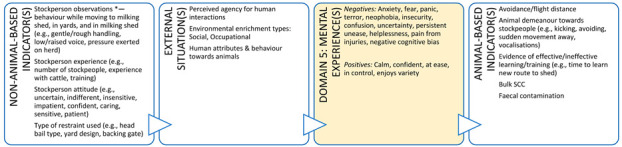

## 5. Proposed Approach to Teaching Animal Welfare Science in Veterinary Curricula and Potential Benefits of This Approach

We propose to reframe veterinary science training to sit within the wider discipline of animal welfare science, that is, training veterinarians as animal welfare experts instead of viewing veterinary science and animal welfare science as either synonymous or as separate entities. The basic premise of our proposed reframing is that veterinary science is an integral part of animal welfare science but that it is not the whole of animal welfare science. The goal of this reframing is to produce veterinarians competent and confident to assess welfare in a more comprehensive manner and in a wider range of situations, thus fulfilling society’s growing expectations of the profession [37]. With this approach, we propose to move the focus of veterinary education and duty of care from Two Domains (Nutrition and Health) towards considering the welfare of animals, and the role of the veterinarian, in all Five Domains.

**Figure 3:**
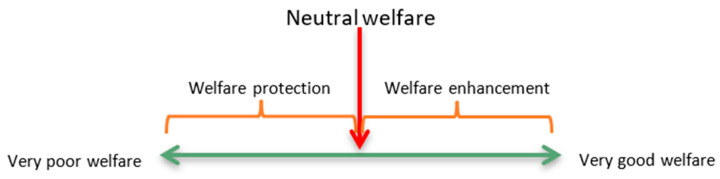
The animal welfare continuum and the roles of veterinarians in protecting (preventing or alleviating negative experiences) and enhancing (promoting positive experiences) on each side of neutral.

There are two key considerations that need to be addressed to allow for this reframing to be achieved. Firstly, veterinarians are currently trained to be experts at recognising, diagnosing, and addressing physical events or states that lead to survival-critical negative mental experiences such as pain, sickness, discomfort, hunger, thirst, breathlessness, and thermal stress. Most welfare indicators used by veterinarians align to physical/functional states in the Nutritional domain (Domain 1) or Health domain (Domain 3), for example, signs of nutritional inadequacy, functional impairment (e.g., lameness examinations), signs of disease, and injuries. The resultant unpleasant experiences of these physical/functional states are critically important, not only to the animal’s survival and productivity but also to its welfare. However, preventing or alleviating such unpleasant experiences will, at best, take an animal along the continuum from some degree of negative welfare state to a neutral state [21]. In other words, simply preventing or rectifying health and production problems does not provide for good welfare or what we might consider a ‘good life’ or a ‘life worth living’ for animals [27]; thus, we might characterise this current role of veterinarians as one of animal welfare ‘protection’ (Figure 3). To provide for good welfare, animals must also have some positive experiences; this is the ‘welfare enhancement’ to which we now aspire and to which veterinarians, if trained appropriately, can make major contributions (Figure 3).

Secondly, we are particularly interested in how to further the use of indicators aligning to Domain 4 (Behavioural Interactions) by veterinarians. The positive affective experiences resulting from conditions (i.e., the animal’s perception of their external situation) in Domain 4 have the potential to enhance an animal’s welfare. This domain is often assessed by considering how well agency is exercised by animals. If animals can fully exercise their agency through the provision of choice and control, their welfare is likely to be evaluated as good.

Veterinarians can enhance animal welfare by recognising impediments to agency and opportunities for improvements that allow animals to exercise their agency. Therefore, to become animal welfare experts, veterinarians must be skilled at assessing and promoting opportunities in Domain 4. By way of example, a veterinarian can make recommendations for how owners might provide opportunities for indoor-only cats to exercise agency (Box 4). If the cat in this situation engages with these opportunities, they are likely to experience positive affective engagement in the form of mental experiences such as playfulness, calmness, and confidence. Therefore, the veterinarian in this example has enhanced the welfare of this cat by assessing its welfare, identifying areas for improvement, and making recommendations that give the cat opportunities to exercise agency, thereby promoting a good life. These discussions can move the veterinarian’s role from one of welfare protection towards promoting welfare enhancement.

In contrast to its current isolated pre-clinical location in veterinary curricula, we propose that animal welfare science should be integrated within all years of veterinary student training programmes. This has several benefits, it: (1) allows for an applied understanding of animal welfare science alongside other related disciplines; (2) encourages integration of student knowledge towards better overall understanding; (3) represents an improved pedagogy for veterinary training; and (4) allows for the role of veterinarians as animal welfare experts to be realised. By incorporating animal welfare science throughout the degree and recognising the need for welfare-centric training, veterinarians will be able to align their veterinary knowledge with an internationally recognised, science-based animal welfare assessment framework; thus, fulfilling their role to society as animal welfare experts.

Box 4 **Companion animal case study.**

**Scenario:** An owner has brought their new kitten to your clinic for its second vaccination. The owner is anxious about the potential impact of their cat on the birdlife in and around their property if they allow it outdoor access. However, they are also of the view that cats need to go outside to lead a full and happy life. The owner has asked you whether they could ensure their cat has a good life as an indoor-only cat.

This case study illustrates how veterinarians can provide animal welfare guidance to owners of indoor-only cats which could enhance the cat’s welfare. A full Five Domains assessment should be performed and discussed with the cat’s owner because there are potential welfare implications of an indoor-only lifestyle in Domains 1 to 3 (e.g., potential for overfeeding leading to obesity and joint disease with resultant experiences of pain and debility). However, for brevity, we have only shown how the discussion could be framed for features collated in Domain 4. Exploration of welfare indicators relating to the animal’s perception of its external situation and the associated mental experiences (Domain 5) is a useful way of framing this discussion.

**DOMAIN 4: Behavioural Interactions (with the Environment)**

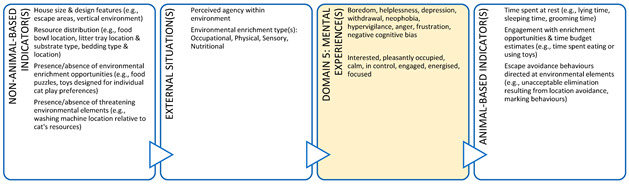

**DOMAIN 4: Behavioural Interactions (with Other Animals)**

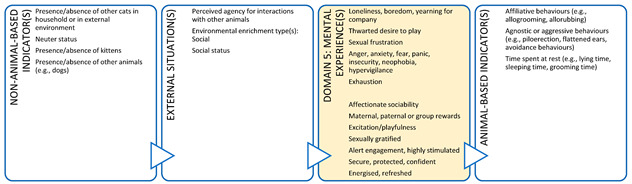

**DOMAIN 4: Behavioural Interactions (with Humans)**

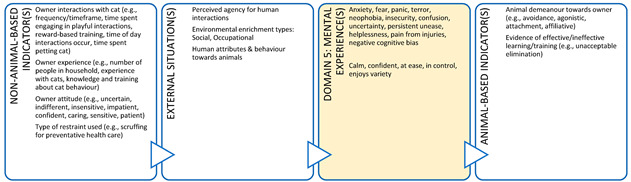

## 6. Conclusions: The Key to Advancing Veterinary Animal Welfare Expertise and Literacy Is through Initial Training and Continued Professional Development

In summary, for veterinarians to be positioned as experts in animal welfare science, they need to first have a holistic and contemporary understanding of what animal welfare is and how it can be scientifically assessed. Veterinarians also need to be motivated to engage with the broader disciplines of animal welfare (science, ethics, policy, and law) and empowered to act as experts in their daily lives. For example, clinical veterinarians need to be able to recognise animal welfare compromise and identify opportunities for welfare enhancement in the animals they care for. The Five Domains Model offers a comprehensive framework for including animal welfare science into veterinary science curricula. Acknowledging the already burgeoning veterinary curriculum, the approach presented here offers a way of integrating animal welfare science across existing curricula without significantly increasing content [37]. This Five Domains approach integrates, reinforces, and reframes animal welfare science in veterinary training to develop welfare literacy [37]. Ethical reasoning skills and knowledge of relevant laws and policies will add to this welfare literacy [64]. Such literacy can then be enacted by aligning this framework with human behaviour change theory [65] and communication skills training [66] to position veterinarians as animal welfare experts.

In this article, we have focused on veterinary training in the first instance to improve animal welfare literacy in the veterinary profession. There are opportunities to advance animal welfare training for veterinarians during their initial education (undergraduate or postgraduate veterinary curricula) and through continued professional development during their veterinary careers. Education is an important step to developing competence, which in turn assists with confidence and workplace satisfaction [67,68]. Appropriate animal welfare training for veterinarians could empower them to act as experts in their daily lives and advance their duty of care from two domains of welfare to all five.

## Figures and Tables

**Table 1:** The range of contexts in which veterinarians are ideally placed to advance animal welfare and examples of the roles they do or could play in each context. Welfare protection refers to preventing or alleviating negative states; Welfare enhancement refers to promoting positive experiences.

Context	Role(s) of Veterinarians
Clinical work	*Welfare protection:* assessing, maintaining, and treating the physical state of animals under their care
	*Welfare enhancement:* encouraging opportunities for animals to engage in behaviours that they find rewarding by influencing and educating people in charge of animals
Expert advice	*Policy & law:* government and industry consult veterinarians for expert advice on new or updated laws and policies that impact animals e.g., Codes of Welfare
	*Media:* expert commentary on animal-focused stories
	*Legal cases:* of animal abuse or neglect
Compliance & monitoring	*Monitoring:* animal welfare verification at slaughter premises
	*Compliance & monitoring:* at rodeo and racing events, and for animals used in research, testing, and teaching
Tertiary education	*Training:* educating next generation of veterinary professionals
